# Scaring as a tool to alleviate crop damage by geese: Revealing differences between farmers’ perceptions and the scale of the problem

**DOI:** 10.1007/s13280-016-0891-5

**Published:** 2017-02-18

**Authors:** Caroline E. Simonsen, Ingunn M. Tombre, Jesper Madsen

**Affiliations:** 1 Department of Bioscience, Aarhus University, Kalø, Grenåvej 14, 8410 Rønde, Denmark; 2 Department of Arctic Ecology, The Fram Centre, Norwegian Institute for Nature Research, P.O. Box 6606, 9296 Tromsø, Norway

**Keywords:** Crop damage, Human–wildlife conflict, Pink-footed goose, Sociological factors, Subsidy

## Abstract

Expanding populations of farmland foraging geese are causing escalating conflict with agriculture. We used questionnaires to investigate farmers´ perceptions in mid-Norway of spring staging geese and the extent to which they attempt to reduce pasture damage by goose scaring. We predicted farmers’ scaring effort (a measure of dissatisfaction) to increase on fields closer to goose roosting sites where goose grazing intensity was highest (measured by dropping counts). Results showed no such relationship, suggesting that farmers’ perception of goose use was not linked to actual goose use, but influenced by sociological factors and individual opinion. These results confirm the need to distribute subsidies/compensation to affected farmers based upon quantifiable measures of goose use rather than complaint levels. To avoid further conflict escalation, it is equally important that managers are aware of farmers´ perceptions and their causes, to effectively target communication about policies and measures to mitigate goose–agriculture problems.

**Electronic supplementary material:**

The online version of this article (doi:10.1007/s13280-016-0891-5) contains supplementary material, which is available to authorized users.

## Introduction

Human–wildlife interactions are increasing worldwide, and managing these can be challenging (Borgerhoff Mulder and Coppolillo 2011; Allen and Garmestani 2015). The application of largely technical solutions, such as deterring, excluding or controlling wildlife, has been attempted to reduce conflicts. But they often fail because the conflicts are rooted in underlying human perceptions of the system and arise from lack of clarity regarding responsibility for managing wildlife problems (Dickman 2010; Redpath et al. 2015). One area of conflict arises from the increase in the population sizes of many western Palearctic goose species (Madsen et al. 1999a; Fox et al. 2010) which has affected a range of human and biodiversity interests (Buij et al. 2017). The main conflicts with agriculture arise due to increasing concentrations of geese foraging on arable crops or grasslands, where they may reduce the grain harvest from cereal fields and grass yields from pastures, which may also necessitate more frequent reseeding (Bjerke et al. 2013; Fox et al. 2016). Consequences for farmers vary between years, crop types, areas and seasons, but certain farmers often suffer disproportionately (Patterson et al. 1989; MacMillan et al. 2004).

Several management solutions have been implemented to alleviate the goose conflict on farmland, often at different scales, levels and with variable success (Fox et al. 2016). A common practice is the establishment of publicly supported schemes via regional or national policies that subsidise farmers’ losses due to goose damage (Owen 1977; van Eerden 1990; Patterson and Fuchs 2001; Kleijn and Sutherland 2003; Cope et al. 2005; Tombre et al. 2013; Eythórsson et al. 2017), although the level of success in alleviating conflicts can be variable (van Eerden 1990; van Roomen and Madsen 1992; Cope et al. 2003; Tombre et al. 2013; Madsen et al. 2014; Koffijberg et al. 2017). Collaborative management initiatives established over larger areas are, in general, more efficient than actions conducted by individual farmers, but for many farmers, the only option is to take personal action. Payment to compensate farmers for goose-related crop loss is not necessarily an acceptable solution, because farmers need all their crops to feed their livestock. Buying alternative foodstuffs may not be an available option. Moreover, a mismatch between available funding and losses can also lead to the situation where farmers instead of applying for compensation are compelled to scare geese off their land using visual and/or audible stimuli (Tombre et al. 2005, 2013). This action may have positive effects for the farmers, because intensive disturbance/scaring over time will interrupt the goose utilisation of a field (Simonsen et al. 2016). As long as there are alternative areas where the geese can forage, scaring geese can be a solution to reduce damage locally.

The Svalbard population of the pink-footed goose *Anser brachyrhynchus* has stopover sites in Norway in spring on their way to the breeding grounds (Madsen et al. 1999b; Tombre et al. 2008). For decades, there have been conflicts between the geese and agricultural interests, because the geese stage at a time when the crops are vulnerable, primarily new-growth pasture but also newly sown cereal fields. A subsidy scheme was introduced in 2006 for the two counties supporting spring stopover sites for the two Svalbard-breeding populations of pink-footed geese and barnacle geese *Branta leucopsis*. The dual objectives were to ensure sites for geese where they can accumulate body fat reserves under undisturbed conditions prior to their onwards migration and breeding while at the same time reducing the agricultural conflict (Eythórsson 2004; Eythórsson et al. 2017). The conflicts between spring staging geese and agriculture were considerably reduced when the subsidy scheme was established (Tombre et al. 2013). Available funding, however, has not been sufficient to provide enough area to support the energy needs of the geese, with the result that the subsidised areas did not balance total crop losses (Baveco et al. 2017). Hence, at some sites in Northern Norway (Tombre et al. 2013) and at the mid-Norway stopover site, Nord-Trøndelag, farmers continue to complain and some farmers continue to scare the geese off their land to protect their crops (Klaassen et al. 2006; Madsen et al. 2014; Simonsen et al. 2016). However, no assessment has been made to determine whether goose scaring is actually necessary or beneficial to the individual farmers or whether it is rooted in the general perception that the presence of geese always equates to damage (i.e. loss of income), and therefore geese should be kept away from the fields. Previous studies have shown that effective goose scaring demands resources. For example, a full-time human bird scarer, systematically and intensively scaring wintering brent geese *Branta bernicla* off arable crops throughout the day significantly reduced goose usage compared to conventional scaring using scarecrows, coloured flags and electronic whistlers (Vickery and Summers 1992). An experimental dosage–response study demonstrated that a person chasing pink-footed geese off pasture fields in Nord-Trøndelag had to make more than two scaring attempts per day which had to be sustained throughout the spring staging period to significantly reduce field use by geese (Simonsen et al. 2016).

In this study, we investigated the degree of scaring activity undertaken by farmers and their motivations for doing so at the goose stopover site in Nord-Trøndelag, Norway. The fact that farmers attempt to scare away geese reflects their dissatisfaction with the prevailing situation for various reasons, which we discuss below, and we use the scaring intensity as a proxy for the level of dissatisfaction. Questionnaires sent out to farmers quantified the scaring effort identified to crop type, the scaring devices used, the farmers’ own arguments for scaring/not scaring geese and their own perception of its effectiveness in terms of reduced goose abundance. In order to investigate if the perception by farmers matched the real use of fields by geese, we quantified goose grazing pressure by systematic counts of droppings in the fields from which we had received responses to the questionnaire.

## Materials and methods

### Study population

The Svalbard-breeding population of pink-footed geese has increased from c. 30 000 in the early 1990s reaching an unprecedented peak of 81 600 individuals in 2012 (Madsen et al. 2015). During spring, the population migrates from Denmark to Svalbard via stopover sites in Nord-Trøndelag in mid-Norway and Vesterålen, northern Norway. Pink-footed geese started to use Nord-Trøndelag as a spring staging area in the late 1980 s, and the region has increasingly attracted more geese (Madsen et al. 1999a). The onset of spring migration has also advanced, a trend associated with the advancement of spring weather (Tombre et al. 2008). At present, the first geese arrive in Nord-Trøndelag in early/mid-April and numbers peak around the first week of May, when almost the entire population is concentrated in the region. The geese depart from Nord-Trøndelag around the middle of May.

### Study area

Nord-Trøndelag is a semi-mountainous region traversed by Trondheim Fjord from northeast to southwest. Sheltered shorelines and large lakes offer roosting sites for the geese, which feed on lowland farmland between urban and forested areas surrounding the fjord and lakes. In April, geese feed on grass pastures, stubble fields and waste potatoes left from the preceding autumn (Chudzińska et al. 2015). When the weather permits, usually around the first week of May, cereals (mainly barley) are sown and geese increasingly switch from pastures to feed on the sown grain and the resulting seedlings in the new-sown fields. In years with a late spring however, geese have started to depart from the area before sowing starts (Chudzińska et al. 2015). The main conflict between geese and agriculture in the region is related to their use of pastures during spring (Bjerke et al. 2013), and this has escalated because of the increasing population size and prolonged staging period (Eythórsson et al. 2017). Each farmer often has several fields, separated by hedgerows or strips of forest and bedrock, some of which are located far from the farm, making it difficult to maintain surveillance for geese in the fields. This adds another layer of challenge to farmers, if they decide to scare geese as an alternative to accepting subsidies.

### Data

**Fig. 1:**
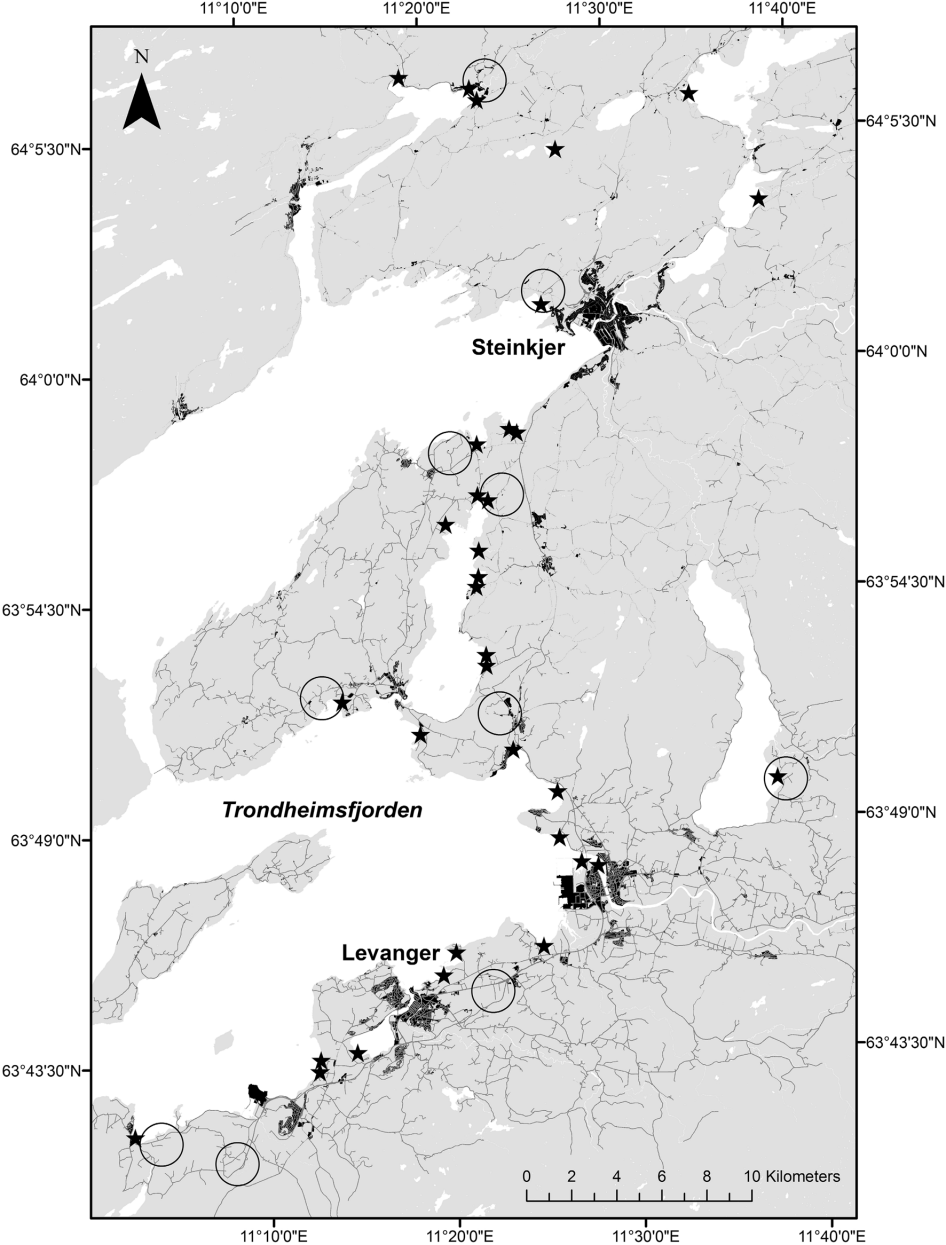
Map of the study area in mid-Norway. *Grey lines* indicate roads, and *black* areas are densely populated areas. *Stars* indicate currently used pink-footed goose roosting sites. The *circles* roughly show areas included in questionnaire survey (see 10.1007/s13280-016-0891-5)

We selected ten randomly stratified areas within the region known to be used by spring staging pink-footed geese. Areas were distributed throughout the Nord-Trøndelag staging area (see Chudzinska et al. 2016), ranging inland from the fjord, each constituting of a well-defined unit with surrounding forests, major roads or water bodies to avoid possible effects of scaring activity in areas bordering the selected sites (Fig. 1). In April 2011, questionnaires were sent to 146 farmers within a 1-km radius of the centre of each area. The questionnaire was sent with a letter describing the objective of the study, explaining that participation was voluntary and anonymous. Questionnaires contained questions regarding how often the person on average scared geese per day, from what type of crops, using what method. If the person answered no to scaring, they were asked the reason for not scaring (see Supplementary material). By the end of the spring staging period (10–11 May), all fields subjected to scaring included in the study area were visited, in addition to fields that were either subsidised or provided ‘no scaring’ responses. As subsidised fields are not subject to scaring (a prerequisite for receiving subsidy), they represented controls for how geese are distributed on fields when not scared from fields along with the ‘no scaring’ replies. We assume that we have a representative target group in the survey as the areas selected were randomly stratified within typical goose areas in spring.

Geese produce droppings every 5 min and droppings remain visible in the vegetation for 3–4 weeks (Madsen 1985); so counts undertaken at the end of the staging episode represented the cumulative use during the main spring staging period of geese. We used a standardised design (see Simonsen et al. 2016) to count goose droppings within three 2-m radius circles using these as a way to express goose use on each field. The three plots were placed in the field centre, as well as two-thirds and one-third of the distance from the nearest source of disturbance, for example the road. Because it turned out that some farmers owned or leased several fields in different areas, but only provided one response, we used data from the field with the highest average dropping density on the respondent’s farm. This was because we expect reported scaring effort to be a response to the field subjected to the highest degree of goose use on his holding.

### Statistics

Relating goose dropping density (as an index of goose use of a field) to the degree of scaring (as an index of farmer dissatisfaction) is not straight forward. Farmers hosting greatest goose densities naturally are likely to scare the most, but such intensified scaring reduces accumulated dropping densities at the end of the season on fields that initially showed highest goose feeding densities. To account for this confounding effect, we used goose dropping counts from subsidised fields and those belonging to farmers who answered “no” to scaring, to create a baseline model to predict goose use in terms of dropping densities and distance to roost in the absence of scaring. We then tested how scaring effort was related to distance to roost. The scaring effort data obtained were divided into four categories; ‘no scaring’, ‘less than twice per day’, ‘twice per day’ and ‘more than twice per day’. Because previous work had shown that goose use of fields declines with distance to roost (Jensen et al. 2008), we hypothesised that (i) goose dropping densities would decline with increasing distance from roost and, hence, (ii) goose scaring effort would similarly decline with increasing distance from roost due to lower goose occurrence. We only used data from grass pastures because the sample size for new-sown cereal fields was too low, given that most farmers had not sown cereals by the time of the survey. We used ArcGIS to measure the distance between each field and the nearest roosting site (see Chudzińska et al. 2016 for an overview of roosts). All statistical analyses were calculated in R version 2.14.0 (R Development Core Team 2013). We applied a generalised linear model with Poisson error term (glm), as well as a negative binomial model (nb.glm) in the package MASS (Venables and Ripley 2002). We used Akaike Information Criterion (AIC) to compare each of our distance-based models to select the model with the best fit. Then, we compared the chosen model with the null model that predicted no change in dropping densities in relation to distance to roost. We then compared the chosen model with the simple model that predicted no change in dropping densities in relation to distance to roost. Models with AIC values differing by more than 2 were considered different, i.e. the lower value expressing the stronger model (Anderson et al. 2000). Finally, we tested how scaring effort was related to distance to roost. We used a Kruskal–Wallis test to test if the distance to roost had identical data distributions across the four scaring effort categories.

## Results

Ninety-six farmers responded to our questionnaires (66%); of those, 62 replies (42%) were completed with all the requested information. Of the 62 replies, 25 reported doing some sort of scaring and 37 not doing so. The 37 ‘no scaring’ fields plus 29 fields that were subsidised were used as controls (*n* = 66) to investigate the goose utilisation of fields not subjected to scaring.

### Crop types, scaring methods and justification for ‘no scaring’

**Fig. 2:**
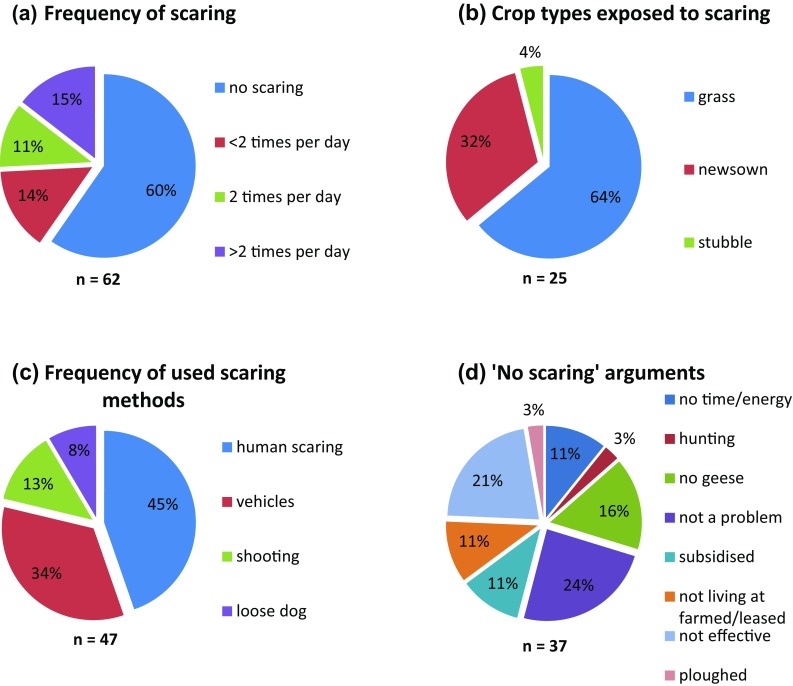
Pie charts showing the distribution of answers (%) from farmers involved in the study with regard to daily frequency of scaring (**a**), crop types exposed to scaring (**b**), scaring methods used (**c**) and arguments for why not scaring geese (**d**). *n* shows sample size (note: several farmers use multiple scaring methods, hence the larger sample size in 1c)

The majority of interviewed farmers (*n* = 62) did not scare geese off their fields (60%, Fig. 2a), whereas 15% of farmers scared geese more than twice per day. The farmers mostly protected the pasture fields (64%) and about one-third protected the new-sown cereal fields (32%) (Fig. 2b). Human scaring and scaring using a vehicle (mostly tractors or all-terrain vehicles) were the most common methods for scaring geese (45 and 34%, respectively, Fig. 2c). Several reasons were equally forthcoming to justify why farmers did not scare geese, with “not a problem” (24%), “not effective” (21%) and “no geese” (16%) as the main arguments (Fig. 2d). Less frequently, the respondents answered that there was no need to scare because they had ploughed (3%) or that the increased possibility of autumn hunting of pink-footed geese was the reason they did not scare them during spring (3%).

### Goose utilisation of fields

**Fig. 3:**
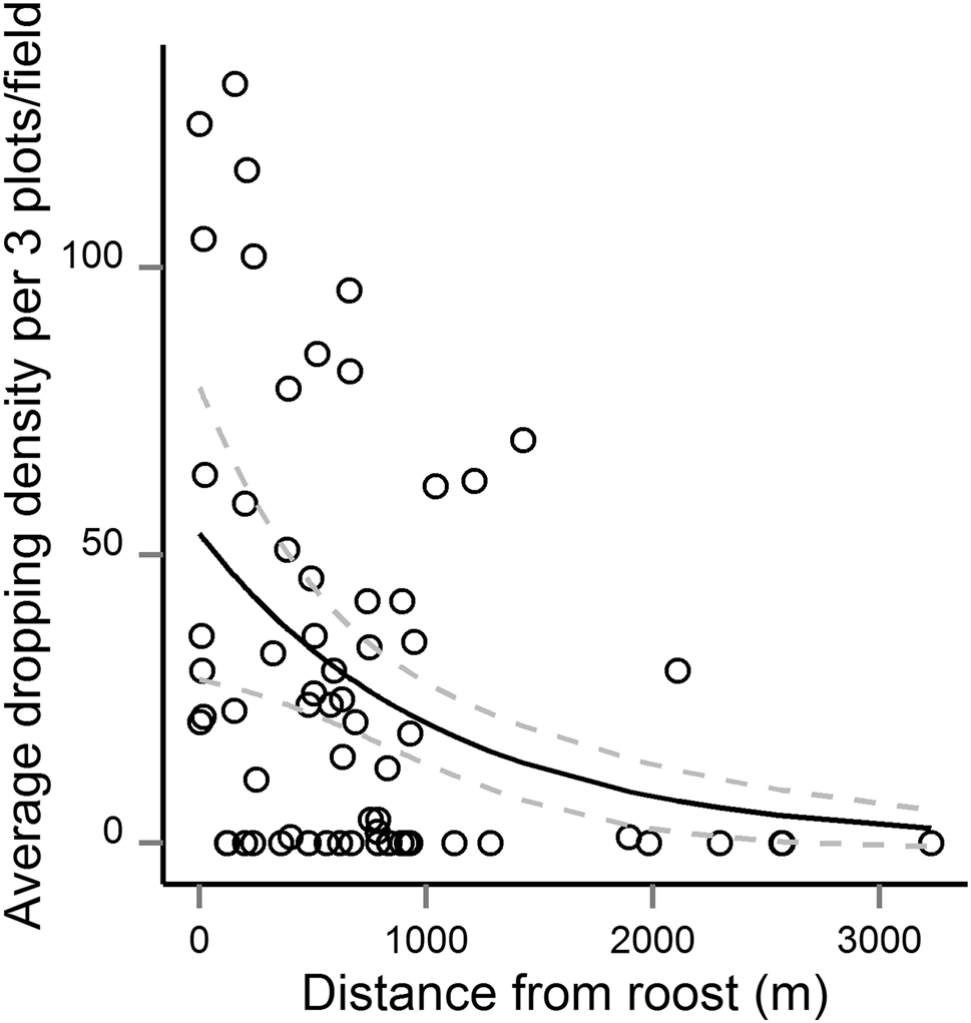
Relationship between goose use expressed by the average dropping density on undisturbed pasture fields and the distance to roost (three plots per field and plot size 12.6 m^2^). The *solid line* is the *fitted line* based on a negative binomial model (nb.glm (droppings ~ distance)), and the *grey dotted lines* indicate the 95% confidence intervals

The negative binomial model (AIC = 563.05) produced the better AIC score for the relationship between distance to roost and the droppings density compared to the GLM model with the Poisson distribution (AIC = 656.24). Comparing the negative binomial model to the model that assumed no relationship between droppings and distance (AIC = 571.31) showed how the negative binomial model fitted the data better according to the difference in AIC (Table 1). Figure 3 illustrates this negative relationship that with a decrease in goose dropping densities, the further away the field was from the roost.

**Table 1:** AIC values comparing models with number of droppings per test circle on grass fields as response variable in relation to distance to roost. The lower AIC indicates the better model if ΔAIC is more than 2.0 (values in bold). The comparison A1 versus A2 compares AIC values from a generalised linear model (glm) with a Poisson distribution to a negative binomial model (nb.glm) which supports the latter as the best fit for the data. Model B1 is the best fit from the A1 versus A2 comparison which is then compared to the B2 model which assumes no positive or negative relationship between the number of droppings and distance to roost

Model	Test (best fit for data)	AIC
A1	glm (droppings ~ distance, Poisson)	656.2
**A2**	**nb.glm (droppings ~ distance)**	**563.1**
	ΔAIC	93.2

Model	Test (best fit vs. no relationship)	AIC
**B1**	**nb.glm (droppings ~ distance)**	**563.1**
B2	nb.glm (droppings ~ 1)	571.3
	ΔAIC	8.3

### Scaring effort

**Fig. 4:**
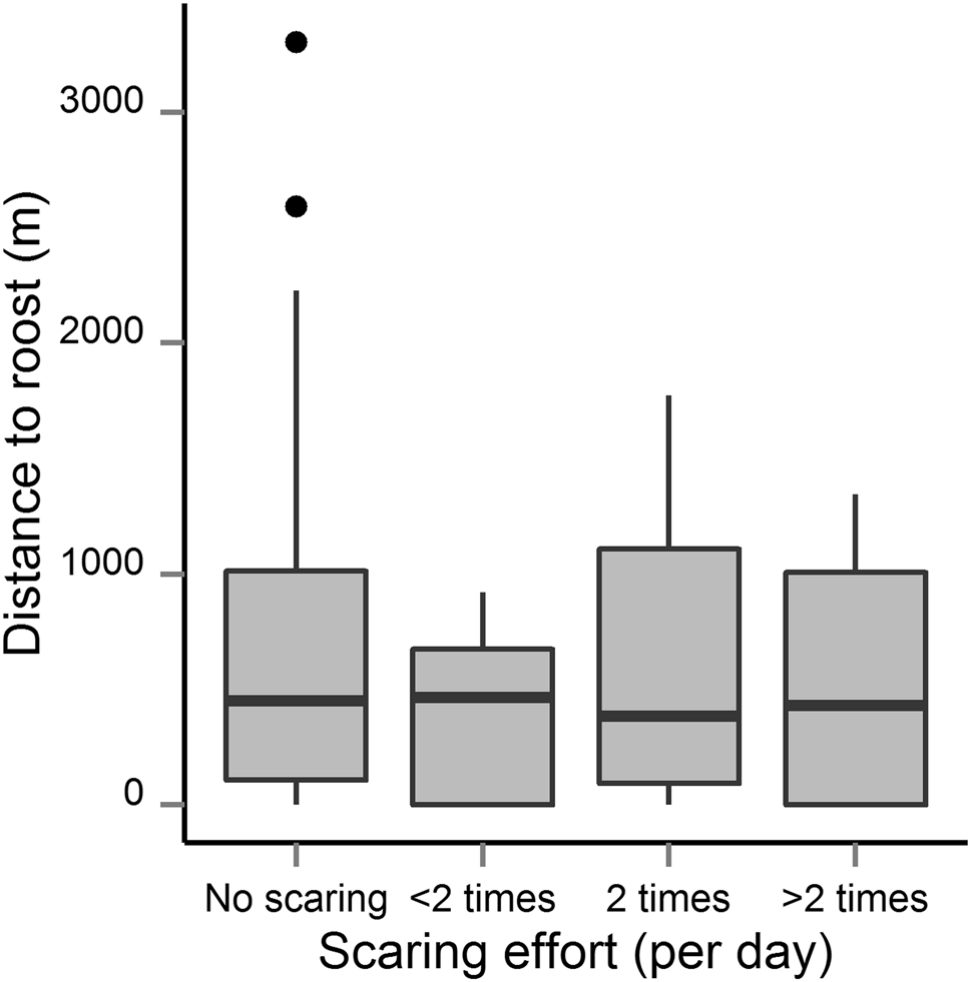
Boxplot showing the relationship between distance to roost and the average scaring effort by farmers on a daily basis. The *solid horizontal black line* in each box indicates the median, the box, the upper and lower 75th and 25th percentiles, respectively, and *black dots* the outliers

There were no significant differences between the scaring effort categories ($\chi_{ 3}^{ 2}$ = 1.18, *P* = 0.76); so we accept the null hypothesis and conclude that the four scaring effort categories did not differ significantly between distance to roost categories (Fig. 4), suggesting that individual farmers did not adjust their scaring effort to actual goose use on their property.

## Discussion

Exclosure studies carried out over several years in Nord-Trøndelag showed that there was a significant relationship between goose grazing intensity (based on goose dropping densities) and yield loss, although with considerable variability in yield loss between years (Bjerke et al. 2013; Bergjord Olsen et al., unpublished results). Hence, we consider dropping counts a good proxy for goose use of specific fields and indicative of direct reductions in subsequent yields. We show that goose grazing intensity declined with distance to goose roosts, which is in accordance with earlier studies (Jensen et al. 2008, 2016; Chudzińska et al. 2016). Hence, it is expected that crop damage will also decrease with distance to roosts. The implication is that scaring of geese more than 1–1.5 km from the roost seems to be unnecessary. Nevertheless, we found that scaring efforts were not related to the distance to roosts.

The majority (85%) of the farmers participating in the current survey reported that they scared geese away twice or less per day. However, as we have shown elsewhere (Simonsen et al. 2016), to be effective, scaring has to be done several times per day, and this does not match with the most frequent level of scaring reported here. Hence, on the majority of farms, the prevailing level of scaring was probably ineffective.

Recently, Fox et al. (2016) reviewed studies on goose management conflicts and found that no complete review of the effects of scaring devices for geese has been conducted. The effectiveness of scaring geese varies, compounded by the use of different methods and devices in different areas and seasons with different species (see Summers and Hillman 1990; Vickery and Summers 1992; Mason and Clark 1995). In the present study, different scaring methods were used, with human scarers, vehicles (usually tractors or all-terrain vehicles) and firing shots over flocks as the most common. Local farmers stated that it was necessary to switch between different scaring methods to have any effect and especially to avoid habituation, supported by experiences from farmers in other regions of Norway (Eythórsson 2004). However, coupled with the fact that the majority of farmers were unaware of the need to be systematic in their frequency of scaring, it seems that the choice of scaring method was also based on personal beliefs rather than documented evidence. Overall, it appears that there is a mismatch between what farmers practice and the level at which there is a real problem with goose damage, as well as understanding what constitutes a deterrent effect for geese.

Farmers gave various reasons for not scaring geese off their property. For those involved in the subsidy scheme, scaring geese was simply not allowed. Forty percent of the farmers also replied that there either were no geese or that the geese were not a problem. On the other hand, some reported that they had no time or energy, or that they thought it was ineffective to scare the geese. These arguments make some sense in the light of the persistence needed to achieve an acceptable level of displacement (e.g. Vickery and Summers 1992; Simonsen et al. 2016).

Managing geese on farmland is a challenging process and requires not only knowledge about the nature of the interactions between the geese and agriculture and the feasible technical means of reducing the damage (e.g. Tombre et al. 2013; Madsen et al. 2014; reviewed in Fox et al. 2016) but also an understanding of the societal context. In the present case, some farmers continue to complain despite an increasing provision of subsidy (Eythórsson et al. 2017). Without doubt, geese cause damage to crops in the area (Bjerke et al. 2013; Bergjord Olsen et al., unpublished data), but as we show in this study, not all the dissatisfaction, expressed through the scaring of geese, is justified in terms of the damage problem. In areas where geese actually do not cause a problem, some of the scaring activity may be carried out under various misconceptions (e.g. the mere presence of geese equates to agricultural damage and the perceived effectiveness of scaring geese on an occasional basis). However, some responses from farmers also suggest that there is a general dissatisfaction with the present management system (e.g. that the authorities are not providing sufficient support), with the result that farmers scare geese for more symbolic reasons, possibly to demonstrate their frustrations (see also Eythórsson et al. 2017). Whatever the reasons, these actions represent a stimulus which keeps fuelling the conflict. On the one hand, our study demonstrates that the distribution of subsidies to affected farmers should be based on quantitative measures of goose use rather than on the basis of individual complaints. On the other hand, in order to avoid an escalation of current conflicts, it is important for managers to be aware of the various perceptions among farmers and their possible underlying causes (see Dickman 2010). At the moment, farmers are scaring geese unnecessarily and managers have to spend time coping with dissatisfied farmers (Eythórsson et al. 2017). In order to get out of this ‘lose-lose’ situation, more resources need to be invested in communication about the issues, such as policy, the subsidy instruments on offer, effectiveness of scaring and the rationale behind the distribution of subsidies to reduce the goose–agriculture problems.

## Electronic supplementary material

Below is the link to the electronic supplementary material.

Supplementary material 1 (PDF 509 kb)
