# Supplementary material for: Scaring as a tool to alleviate crop damage by geese: Revealing differences between farmers’ perceptions and the scale of the problem
Source: Ambio. 2017 Feb 18;46(Suppl 2):319–27. doi: 10.1007/s13280-016-0891-5 (PMC5316329; doi:10.1007/s13280-016-0891-5)
Supplement: Supplementary file 1 — Supplementary material 1 (PDF 509 kb) [file 13280_2016_891_MOESM1_ESM.pdf]

*Ambio*

Electronic Supplementary Material

This supplementary material has not been peer-reviewed

Title: **Scaring as a tool to alleviate crop damage by geese: Revealing differences between farmers' perceptions and the scale of the problem**

Authors: Caroline E. Simonsen, Ingunn M. Tombre, Jesper Madsen

**Supplementary material – Questionnaire**

<table>
<tr><td colspan="2">Date:</td><td colspan="4"></td></tr>
<tr><td colspan="2">Name and address:<br>(will be treated confidentially)</td><td colspan="4"></td></tr>
<tr><td colspan="6">Encircle your choice</td></tr>
<tr><td>1)</td><td>Do you scare pink-footed geese from your fields during April and May?</td><td>YES</td><td>NO</td><td colspan="2"></td></tr>
<tr><td colspan="6">If YES, continue to question 2, if NO continue to question 5</td></tr>
<tr><td>2)</td><td>How often do you scare foraging geese off your fields?</td><td>More than two times per day</td><td colspan="2">Two times per day</td><td>Less than two times per day</td></tr>
<tr><td rowspan="2">3)</td><td rowspan="2">Which scaring methods do you use? (feel free to mark several methods if necessary)</td><td>Approaching on foot</td><td>Clapping</td><td>Shouting</td><td>Dog</td></tr>
<tr><td>Tractor Car</td><td>ATV</td><td>Shooting</td><td>Flaregun</td></tr>
<tr><td>4)</td><td>Choose which crop you primarily protect by scaring geese</td><td>Grass pasture</td><td colspan="2">Stubble field</td><td>Newsown cereal</td></tr>
<tr><td colspan="6">The following is only relevant if you answered NO to scaring geese in question 1</td></tr>
<tr><td rowspan="4">5)</td><td rowspan="4">What is the main reason you do not scare geese?</td><td colspan="2">There are no geese on my fields</td><td colspan="2">I get subsidy/compensation from the municipality</td></tr>
<tr><td colspan="2">I do not think they cause a problem</td><td colspan="2">I do not think scaring is effective</td></tr>
<tr><td colspan="2">I do not have the time</td><td colspan="2">I hunt during autumn and would like to see them return</td></tr>
<tr><td colspan="4">Other reasons:</td></tr>
<tr><td colspan="2">Feel free to add additional comments:</td><td colspan="4"></td></tr>
</table>
